# Association of the JAZF1 Variant in Adults With a Parental History of Type 2 Diabetes Mellitus In Pakistan

**DOI:** 10.7759/cureus.11930

**Published:** 2020-12-06

**Authors:** Sheh Zano, Zil E Rubab, Saeeda Baig, Moazzam A Shahid, Farah Ahmad, Faizan Iqbal

**Affiliations:** 1 Biochemistry, Ziauddin University, Karachi, PAK; 2 Community Health Sciences, Ziauddin University, Karachi, PAK; 3 Orthopaedics, Patel Hospital, Karachi, PAK

**Keywords:** type 2 diabetes mellitus, multifactorial inheritance, jazf1, family history, genetic variant, systemic, pakistani population

## Abstract

Background

Type 2 diabetes mellitus (T2DM) is a chronic multifactorial condition and quickly growing disease in Pakistan. Many genes together with Zinc finger protein 1 (JAZF1) have already been described earlier in the literature but the role of JAZF1 in this subset of the population is yet to define. This study was aimed at identifying JAZF1 polymorphism and the risk of developing T2DM in persons with a parental history of T2DM in the Pakistani population.

Methods

DNA samples from 75 non-diabetic Pakistani participants with a family history of T2DM and 75 controls were evaluated by using a polymerase chain reaction (PCR) and the restriction fragment length polymorphism method.

Results

The alleles AA and AG and the GG genotype of JAZF1 (rs864745) varied considerably in frequency distribution between cases and control (p<0.05). The GG was independently and significantly associated with cases who had a family history of T2DM [odds ratio (OR) 2.6 (95% confidence interval (Cl) 1.3-5.1); p=0.005] while the AA allele was significantly associated with controls without a family history of T2DM [odds ratio (OR) 0.39 (95% confidence interval (Cl) 0.2-0.7); p=0.0059] and the allele AG has no significance and was equally distributed among control and cases with p-value=1.000.

Conclusion

Genotype GG of the JAZF1 variant was found significantly associated with the risk of developing type 2 diabetes mellitus in the Pakistani subset of the population.

## Introduction

Type 2 diabetes mellitus (T2DM) is a systemic disease that is indicated by increasing blood sugar levels and is secondary to defect either in insulin production or insulin resistance [1]. Diabetes mellitus is a primary health concern in Pakistan [2-4]. Previously, the prevalence of type 2 diabetes mellitus was related to an abundance of wealth, urbanization, and a sedentary lifestyle. Type 2 diabetes mellitus is more prevalent in middle and low-income countries like Pakistan [5-7]. According to the World Bank’s country classification, Pakistan is a lower-middle-income country [8]. Pakistan is the sixth most densely populated country in the world and the prevalence of type 2 diabetes mellitus is still underestimated [9]. The International Diabetes Federation (IDF) reported that the overall prevalence of T2DM in Pakistan was 6.8 % in patients aged between 20 and 79 years [10]. Previous studies also show some disagreement about the results reported by the IDF, and they found that the prevalence of T2DM is found to be 7.2% to 19.2% in different regions of Pakistan [11].

Besides lifestyle and environmental risk factors, type 2 diabetes mellitus also has an established genetic predisposition [12]. Risk factors, reported by multiple studies in the development of type 2 diabetes mellitus, include obesity, hypertension, hypercholesterolemia, along with a parental history of T2DM [13].

JAZF1 encodes a putative transcription factor that interacts with protein NR2C2 (nuclear receptor subfamily 2, group C, member 2 - also referred to as TR4 orphan nuclear receptor) and inhibits transactivation that targets many essential genes in metabolism. Impaired function of beta cells has been correlated with JAZF1 locus variants as a gene transcriptional repressor that negatively influences the metabolism of glucose, which emphasizes that these susceptibility alleles may result in a decreased transcription of JAZF1. Such mutants like rs1635852, rs849133, and rs849142, rs1635852, and 864745 have already been linked with T2DM [13]. SNP rs864745 has been found to be more prevalent in our population; therefore, it is considered in our study [14].

However, not only are the data regarding the association of the rs864745 variant of JAZF1 limited but also its relationship with parental history is not established. The purpose of this case-control study was to identify the polymorphism of the JAZF1 variant (rs864745) as an additional threat of developing T2DM, especially in patients who had a parental history of T2DM.

## Materials and methods

Patient selection

The sample size was calculated by using OpenEpi software (www.OpenEpi.com) with a two-sided confidence level (1-alpha) 95 and power of 80 with a hypothetical proportion of cases with exposure 24.56. A total of 150 participants (75 non-diabetics with a parental history of T2DM and 75 healthy controls with no parental history) belonging to the same demographic features were enrolled. The diagnosis of T2DM was based on World Health Organization (WHO) guidelines (fasting glucose levels as low as 126 mg/dl or as low as 7.0 mmol/L). All consenting participants were given a generalized questionnaire, including demographics and past medical history. Excluded from this study were those patients with comorbidities such as type 2 diabetes and type 1 diabetes. Control subjects (n = 75) with the usual fasting blood sugar (FBS) value were identified from the age and gender-based general population.

Five ml of fasting blood samples were taken from subjects, 3 mL was taken in ethylenediaminetetraacetic acid (EDTA) tubes for molecular analysis, and the other 2 ml in a gray-top tube for FBS. After the extraction of deoxyribonucleic acid (DNA) and FBS analysis, the samples were stocked at -80°.

Clinical and chemical assessments

Clinical and anthropometric parameters were recorded, including weight (kg), height (m), blood group, ethnicity, family history, marital status duration of diabetes Body mass index (BMI). Fasting blood glucose (FBG) is measured using a Glucose- GOD-PAP enzymatic colorimetric method.

DNA extraction

Whole blood was used to extract deoxyribonucleic acid (DNA) (Genomic) using a DNA isolation kit (GeNet Bio Prime Prep^TM^ Genomics, Korea) by adding 20 ul proteinase K solution in an Eppendorf tube. After that, 200 ul of blood was added to it and then 200 ul GB buffer. This was then vortexed for 15 sec, incubated at 56°C for 10 min, and 200 ul absolute ethanol was then added and vortexed for 15 sec. We carefully transferred the lysate into the upper reservoir of the spin column, centrifuged it at 8000 rpm for 1 minute, and then transferred the spin column to a new collection tube for filtration. Five-hundred ul of buffer GW1 was added to the spin column and centrifuges at 8000 rpm for one min. After which, the flow-through was discarded and the spin column was transferred to a new collection tube. Five-hundred ul of the GW2 buffer was added to the spin column and centrifuged at 8000 rpm for 1 min, after which the flow-through was discarded. The spin-column was reassembled with its collection tube, centrifuged one more time at 12000 rpm for one to two min to completely remove ethanol. Finally, the spin column was transferred to a new tube for elusion. Then, 200 ul GE buffer was added into the spin column. After waiting for 1 min at room temperature and centrifuging at 8000 rpm for one min, the flow-through was collected and stored at −80 °C. Nanodrop and gel electrophoresis was used to evaluate the quantity and quality of DNA.

Analysis of genotyping polymorphism by polymerase chain reaction (PCR) and restriction fragment length polymorphism (RFLP)

JAZF1 polymorphism for 378 bp DNA fragments was done by polymerase chain reaction by using the following primer:

forward (A > G) 5′-GAGCCATATAAGTGATGCTCAAA-3′ (Alharbi KK et al., 2015 [15])

reverse 5′-GGTTGTCAGGCTTTCCATGT-3′ (Alharbi KK et al., 2015 [15])

PCR was programmed with an initial denaturation at 95 °C for five minutes, followed by 40 cycles of denaturation at 95°C for 30 sec, annealing at 58 °C for 30 sec, and extension at 72 °C for 40 sec. PCR was then followed by RFLP in which restriction endonuclease (Thermo Scientific #ER0771; Waltham, Massachusetts) 2 UL SSPI was used to digest the 10 µL PCR product, 18 ul nuclease-free water, and 10x buffer G mix gently, and this was incubated at 16 h at 37 °C for A allele (378bp), for G allele (338 and 40-bp), and AG allele (378 bp, 338 bp, 40 bp). Gel electrophoresis was used to visualize in which 1-2 gm of agarose was used according to the need in each gel, with a voltage of 110 amp for 40 minutes.

Statistical analysis

The Statistical Package for the Social Sciences (SPSS) version 25.0 (IBM Corp., Armonk, NY) was used for the analysis of data. Data regarding numerical variables are mentioned as mean ± standard deviation. Chi-square was used as the test of significance for genotype frequency between cases and controls for each single nucleotide polymorphisms (SNPs). For inter and intragroup comparison of genotype with age, BMI, and FBS between cases and controls. Analysis of variance (ANOVA) was applied as the test of significance. Bivariate analysis was carried out for finding the association of genes with cases and controls and was reported as an odds ratio. p-value <0.05 was considered significant at a 95% confidence interval.

## Results

The demographic characteristics of the study participants listed in Table 1 show the most participants of our study were in the age group of 18-23 with a frequency of 57 (38%). Gender was equally distributed in our study. Urdu speaking ethnicity was predominant (53; 35.3%) while further results are elaborated in Table 1.

**Table 1:** Demographic data of Pakistani participants

VARIABLES	CONTROL	CASE
Age		
18-23	33 (44%)	24 (32%)
24-28	20 (26.7%)	29 (38.7%)
29-33	14 (18.7%)	12 (13.3%)
34-38	8 (10.7%)	10 (13%)
Gender		
Male	35 (46.7%)	40 (53.3%)
Female	40 (53.3%)	35 (46.7%)
Marital status		
Single	36 (48%)	32 (42.7%)
Married	39 (52%)	43 (57.3%)
Ethnicity		
Sindhi	10 (13.3%)	8 (10.7%)
Punjabi	11 (14.7%)	26 (34.7%)
Pathan	9 (12.0%)	4 (5.3 %)
Baloch	5 (6.7%)	2 (2.7%)
Urdu speaking	27 (36.0%)	26 (34.7%)
Other	13 (17.3%)	9 (12%)

In Table 2, we determine the association between the JAZF1 variant and T2DM risk factors by using analysis of variance (ANOVA) after controlling for threats like age, BMI, and FBS. All the above risk factors are not significant in our study as the p-value of all three risk factors is >0.05.

**Table 2:** Quantitative variables according to the genotype of JAZF1 polymorphism BMI: body mass index; FBS: fasting blood sugar

AGE	CONTROL (Mean+-SD)	CASES (Mean+-SD)
JAZF1 (rs864745)		
AA	25.97+-4.70	27.03+-4.42
AG	26.85+-5.39	26.14+-4.37
GG	25.14+-3.8	26.28+-4.31
P-value	0.644	0.756
BMI	CONTROL (Mean+-SD)	CASES (Mean+-SD)
JAZF1 (rs864745)		
AA	24.90+-4.11	24.7+-3.35
AG	22.62+-3.32	26.75+-5.6
GG	24.07+-4.79	24.5+-4.0
P-value	0.375	0.412
FBS	CONTROL (Mean+-SD)	CASES (Mean+-SD)
JAZF1 (rs864745)		
AA	79.82+-10.45	85.56+-11.33
AG	73.85+-12.78	77.57+-14.03
GG	81.42+-12.33	82.52+-11.14
P-value	0.306	0.221

**Figure 1:**
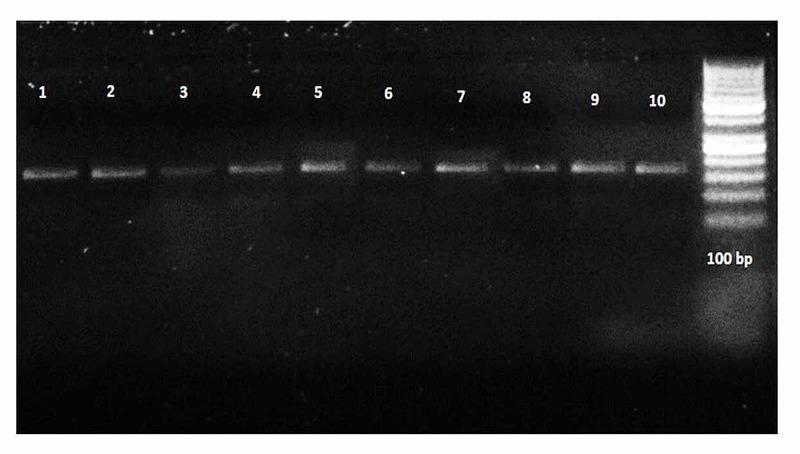
PCR results of the JAZF1 gene (rs864745) Lane 1-10: showing the 378 bp band of the rs864745 JAZF1 gene; Lane 11: showing the 100 bp ladder PCR: polymerase chain reaction

**Figure 2:**
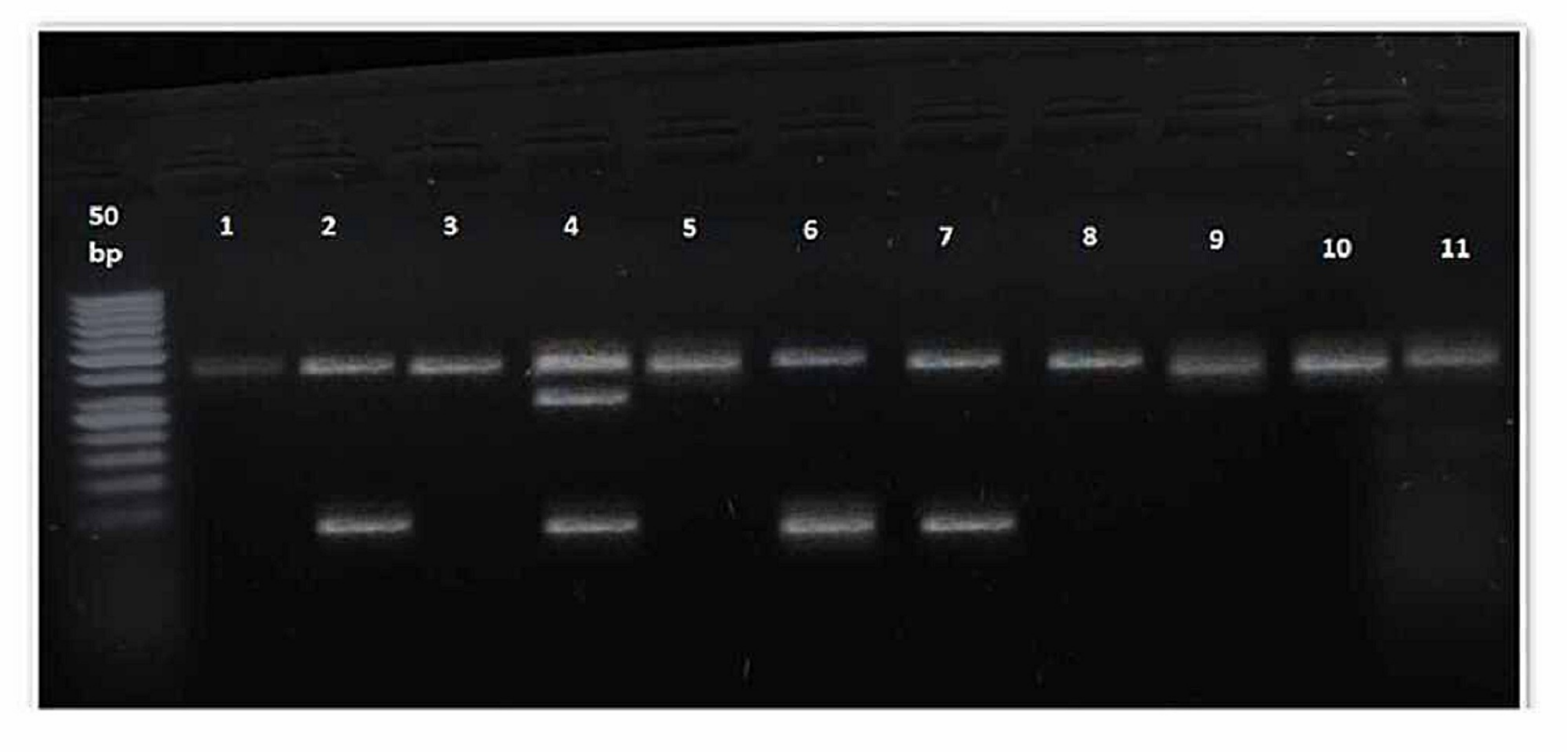
RFLP results of the control group of the JAZF1 gene (rs864745) Lane 1, 3, 5, 8, 9, 10, and 11: showing wild type AA genotype (378 bp); Lane 2, 6, and 7 showing a homozygous mutant GG genotype (338 and 40); Lane 4 showing a heterozygous AG genotype (378, 338, and 40 bp). The left side of the picture showing a 50 bp ladder. RFLP: restriction fragment length polymorphism

**Figure 3:**
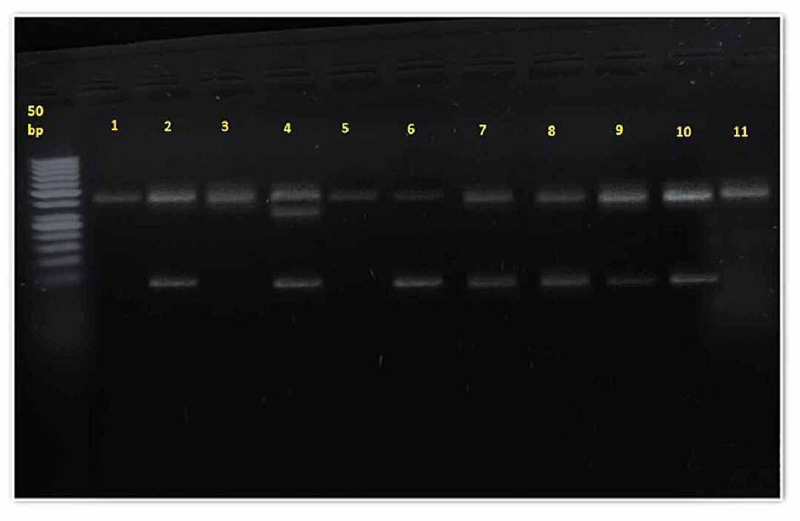
RFLP results of the cases group of the JAZF1 gene (rs864745) Lane 1, 3, 5, and 11: showing wild type AA genotype (378 bp); Lane 2, 6, 7, 8, 9, and 10 showing a homozygous mutant GG genotype (338 and 40); Lane 4 showing a heterozygous AG genotype (378, 338, and 40bp). The left side of the picture showing a 50 bp ladder. RFLP: restriction fragment length polymorphism

Table 3 displays the genotype distribution for rs864745 among people with a family history of type 2 diabetes and control subjects without a parental history of type 2 diabetes mellitus. The distribution of the AA, AG, and GG genotypes (p<0.005) varied significantly between cases and control. Similarly, the frequency of AA (rs864745) JAZF1 was significantly more in the control than in cases (62.6% vs 40%), whereas the occurrence of the AG genotype is not significant in both groups (9.3% vs 9.3%) while the GG genotype was considerably more in cases than in controls (28% vs 50.6%). Detailed results are elaborated on in the table. Figures 1-3 show the PCR and RFLP results of the JAZF1 gene (rs864745).

**Table 3:** Genotype distribution of JAZF1 variant (rs864745) in the case and control groups

JAZF1	CONTROL	CASE	Odd ratio	p.value	C/I
AA	47(62.6%)	30(40%)	0.39	0.0059	0.2-0.7
AG	7(9.3%)	7(9.3%)	1.00	1.000	0.3_3.0
GG	21(28%)	38(50.6%)	2.6	0.005	1.3-5.1

Table 3 displays the genotype distribution for rs864745 among people with a family history of type 2 diabetes and control subjects without a parental history of type 2 diabetes mellitus. The distribution of the AA, AG, and GG genotypes (p<0.005) varied significantly between cases and control. Similarly, the frequency AA (rs864745) JAZF1 was significantly more in the control than in cases (62.6% vs 40 %), whereas the occurrence of the AG genotype is not significant in both the groups (9.3% vs 9.3%) while the GG genotype was considerably more in the cases than the controls (28% vs 50.6%). Detailed results are elaborated in Table 3.

## Discussion

The purpose of this case-control study is to evaluate the association of the JAZF1 (rs864745) variant and the development of T2DM, especially in Pakistan. Alharbi et al. conducted a study in the Saudi population with 400 T2DM and 400 healthy subjects [15]. They found that the JAZF1 (rs864745) variant is frequently associated with T2DM with an increased waist circumference. This study was conducted in patients who were diabetic and had a parental history of diabetes. The findings of this study were similar in the sense that JAZF1 is associated with T2DM but in our study, we include non-diabetic individuals (cases) with a parental history of T2DM, which is in contrast to the study conducted in the Saudi population who were diabetic. They also found that the AG+GG genotypes are frequently associated with increased waist circumference and fasting blood glucose levels. We also found the GG genotype in non-diabetic patients who had a parental history of T2DM. Stancakova et al. conducted a study at Kuopio University Hospital, Finland, in non-diabetic Finnish men [16]. They actually failed to confirm an association with the JAZF1 (rs864745) variant.

Rees et al. conducted a case-control study in two Punjabi populations, predominantly originating from Mirpur Khas, Pakistan [17]. The authors concluded that 13 genes are associated with an increased risk of T2DM in Pakistan. On the contrary, our study includes individuals with different ethnicity and with a parental history, which might be helpful to identify high-risk individuals in different regions of our country and for early diagnosis. To the best of our knowledge, this study is the first from a single center of Karachi, Pakistan, to report the association of the JAZF1 variant and T2DM. Despite the increasing prevalence of T2DM in our country, we still lack agreement to make any firm consensus between the JAZF1 (rs864745) variant and the development of T2DM. Therefore, this study is conducted to highlight individuals (cases) with a parental history of diabetes who are at risk of developing T22DM, as early detection prevents long-term consequences of T2DM [18].

A number of studies have already been published to report results regarding the association of the JAZF1 (rs864745) variant and T2DM, especially in well-developed countries, but more studies are needed to use this gene as a biomarker [19]. In our study, the genotype distribution of the JAZF1 variant (rs864745) differs among cases and control subjects. We found the GG genotype more in patients who had a parental history of T2DM, whereas the AA genotype was found more commonly in control subjects. We found a statistically significant relationship between these two genotypes, as shown in Table 3 with P-value <0.05. The AA genotype is not found to be statistically significant in our study. Based on these findings, patients that have the GG genotype are more prone to develop type 2 diabetes mellitus. More support for the possible association between the JAZF1 (rs864745) variant and T2DM, confounding factors were evaluated between case and control subjects to identify the genotype responsible for the development of T2DM, especially in patients who had a parental history of diabetes as shown in Table 2.

Previous studies had already demonstrated the association of T2DM with body fat and BMI [20-23]. In our study, patients who had a parental history of T2DM had normal BMI and body fat distribution. Therefore, the exact association between T2DM with body fat distribution and BMI is not well-elucidated in the current study.

The present study showed that compared with normal subjects (controls), we found the GG genotype in cases that had a parental history of type 2 diabetes mellitus. The JAZF1 variant (rs864745) commonly resides in intron 1, which actually encodes a transcriptional repressor of nuclear receptor subfamily 2, the group C member 2 (NR2C2) gene. NR2C2 widely displays a phenotype of growth retardation, hypoglycemia, and reduced glycogenesis by decreasing the activity of PEPCK. The role of pancreatic beta-cell function has not been demonstrated yet. However, one must hypothesize that the JAZF1 variant (rs864745) also affects beta-cell mass and function [24-26]. Kobiita et al. conducted a study regarding the role of JAZF1 in the homeostatic control of ribosome biogenesis and function in metabolic stress. They found that SNP rs1635852 located in intron X of JAZF1 is a key factor in pancreatic beta-cell for mediating metabolic stress signals and regulating protein translation in response to increased demand for insulin [27].

Previous studies already establish the association of environmental, metabolic, and genetic roles in the development of T2DM [28-30]. Our study is unique in the sense that all cases were non-diabetic and they only had a family history of T2DM. This study highlights the subjects who are at a high risk of developing T2DM in the future. The early recognition of high-risk patients by genetic means prevents patients from the long-term consequences of T2DM. The prevalence of T2DM in a developing country like Pakistan is increasing rapidly. Therefore, this study was conducted to determine a genotype of the JAZF1 variant rs864745 in the early detection of patients who had a parental history of T2DM.

The limitation of our study is that it is conducted in a single center; therefore, the result of this study cannot be generalized for the entire population. Second, the sample size of our study is small, which compromises our findings in order to establish an association of JAZF1 in adults with a parental history of T2DM; large sample size is required. As the diagnosis of T2DM is a social stigma, and individuals suffering from it have to face lifetime problems; therefore, more studies are needed to confirm the above findings and consider this gene as a biomarker in the future.

## Conclusions

Our study concluded that genotypic variants of JAZF1 rs864745 are important to identify individuals at risk of developing T2DM, and it may assist in early recognition of T2DM. People with genotype GG are at increased risk of developing type 2 diabetes mellitus in the future as compared to people with genotypes AA and AG in the Pakistani community.

## Appendices

**Table 4:** SPSS data for results SPSS: Statistical Package for the Social Sciences; BMI: body mass index

SAMPLE	AGE	GENDER	WEIGHT	HEIGHT	BMI	BLOOD GROUP	MARRITAL STATUS	ETHNICITY	FAMILY HISTORY	FBS	JAZF1
cases	29-33	female	70	5.5	25	B+	married	punjabi	yes	93	gg
cases	18-23	male	72	5.9	22.9	B+	single	punjabi	yes	99	gg
cases	18-23	female	46	5.3	16.8	B+	single	punjabi	yes	80	gg
cases	18-23	female	59	5.4	22.3	B+	single	urdu speaking	yes	96	gg
cases	18-23	female	62	5.6	21.3	A+	single	punjabi	yes	97	gg
cases	29-33	female	63	5.6	21.3	B+	married	urdu speaking	yes	91	gg
cases	18-23	female	68	5.7	22.2	B+	single	urdu speaking	yes	80	gg
cases	18-23	male	58	5.8	18.5	A+	single	urdu speaking	yes	100	gg
cases	18-23	female	65	5.4	23.3	B+	single	others	yes	98	gg
cases	18-23	female	55	5.4	19.7	B+	single	urdu speaking	yes	84	gg
cases	18-23	female	51	5.2	19.6	O+	single	baloch	yes	99	AA
cases	24-28	male	68	5.8	21.3	O+	married	sindhi	yes	98	AA
cases	24-28	female	60	5.5	20.8	B+	married	sindhi	yes	81	AA
cases	34-38	female	80	5.6	27	O+	married	urdu speaking	yes	77	gg
cases	24-28	female	52	5.1	20.1	O+	married	urdu speaking	yes	98	gg
cases	34-38	female	80	5.7	26.2	AB+	married	punjabi	yes	63	gg
cases	24-28	male	85	5.6	28.4	AB+	married	pathan	yes	56	gg
cases	18-23	female	78	5.1	26	O+	married	urdu speaking	yes	84	Ag
cases	18-23	male	78	5.1	26	AB+	single	urdu speaking	yes	79	Ag
cases	34-38	male	85	5.11	25.9	0-	married	urdu speaking	yes	64	AA
cases	34-38	female	82	5.7	26.6	B+	married	urdu speaking	yes	65	gg
cases	24-28	male	103	5.8	31.9	B-	married	urdu speaking	yes	77	gg
cases	24-28	female	70	5	28.1	0-	married	others	yes	78	Ag
cases	18-23	male	75	5.4	26.4	A-	single	others	yes	85	Ag
cases	34-38	female	82	5.8	25.4	A+	married	urdu speaking	yes	92	Ag
cases	29-33	female	85	5.6	28.2	A-	married	punjabi	yes	88	gg
cases	29-33	female	80	5.8	25.1	O+	married	urdu speaking	yes	91	gg
cases	24-28	female	80	5.8	25.1	B+	married	urdu speaking	yes	76	gg
cases	24-28	female	53	5.4	18.7	B+	married	urdu speaking	yes	59	gg
cases	24-28	female	59	5.1	17.5	O+	married	sindhi	yes	77	gg
cases	24-28	female	65	5.5	23	O+	married	baloch	yes	83	gg
cases	29-33	female	48	5.4	18	AB+	married	punjabi	yes	77	Ag
cases	24-28	female	90	5.12	37.4	A-	married	others	yes	48	Ag
cases	24-28	male	68	5.11	28.2	B+	single	sindhi	yes	93	gg
cases	18-23	male	60	5.9	19.5	B-	single	punjabi	yes	87	gg
control	18-23	female	53	5.3	20.5	B+	single	urdu speaking	no	95	AA
control	18-23	female	54	5.8	17.6	O+	single	urdu speaking	no	98	AA
control	18-23	female	58	5.7	20	O+	single	urdu speaking	no	82	AA
control	24-28	male	62	5.8	20.7	O+	married	baloch	no	82	AA
control	18-23	male	72	5.8	24.1	B+	single	punjabi	no	85	AA
control	18-23	male	67	5.1	21.1	A+	single	urdu speaking	no	93	AA
control	18-23	male	63	5.9	20.5	O+	single	urdu speaking	no	85	AA
control	18-23	female	57	5.2	22.9	O+	single	pathan	no	84	AA
control	18-23	male	62	5.11	19	O+	single	punjabi	no	82	AA
control	18-23	female	74	5.3	28.9	B+	married	urdu speaking	no	71	AA
control	29-33	female	67	5.2	26.9	O+	married	others	no	77	AA
control	18-23	female	57	5.2	22.9	A+	single	sindhi	no	92	gg
control	18-23	male	100	6.1	23	O+	single	baloch	no	91	gg
control	18-23	female	55	5.4	20.8	B+	single	punjabi	no	64	Ag
control	18-23	male	73	5.9	23.7	B+	single	urdu speaking	no	99	gg
control	18-23	female	55	5.5	20.1	B+	single	others	no	86	gg
control	18-23	female	56	5.8	18.7	B+	single	punjabi	no	80	Ag
control	18-23	female	55	5.3	21.4	B-	single	sindhi	no	90	AA
control	18-23	male	75	5.11	23	A-	single	urdu speaking	no	85	AA
control	29-33	female	90	5.6	32	B+	married	punjabi	no	81	AA
control	34-38	female	80	5.6	28.4	B+	married	others	no	82	AA
control	18-23	female	50	5.7	17.2	AB-	single	urdu speaking	no	85	gg
control	18-23	male	97	5.11	29.8	B+	single	urdu speaking	no	72	gg
control	24-28	male	49	6	14.5	O+	married	urdu speaking	no	85	gg
control	18-23	male	75	5.4	28.3	B+	single	urdu speaking	no	67	gg
control	18-23	male	55	5.7	18.9	AB-	single	urdu speaking	no	79	gg
control	18-23	female	64	5.4	24.2	0-	single	others	no	74	gg
control	24-28	female	55	5.5	20.1	B-	married	punjabi	no	54	Ag
control	24-28	male	55	5.6	19.5	AB-	married	others	no	79	AA
control	18-23	female	55	5.6	19.5	0-	single	urdu speaking	no	78	AA
control	29-33	female	102	5.4	38.5	B+	married	others	no	79	AA
control	18-23	male	55	5.6	19.5	B+	single	urdu speaking	no	63	AA
control	18-23	male	90	5.1	28.4	AB-	single	urdu speaking	no	69	AA
control	34-38	female	74	5.7	25.5	O+	married	others	no	64	AA
control	24-28	male	85	5.9	27.6	B-	married	urdu speaking	no	74	gg
control	18-23	female	59	5.7	20.3	AB+	single	others	no	98	gg
control	18-23	male	63	5.8	21	B+	single	urdu speaking	no	77	gg
control	34-38	female	80	5.5	29.3	AB+	married	urdu speaking	no	89	gg
control	29-33	female	70	5.6	24.9	B+	married	urdu speaking	no	86	gg
control	29-33	female	73	5.4	27.6	AB+	married	sindhi	no	98	gg
control	24-28	male	72	5.6	25.5	0-	single	others	no	67	gg
control	29-33	female	85	5.5	31.1	AB+	married	punjabi	no	68	gg
control	29-33	female	85	5.3	33.1	B+	married	urdu speaking	no	73	gg
control	29-33	female	58	5.5	21.1	B+	married	urdu speaking	no	100	AA
control	29-33	female	58	5.5	21.1	AB+	married	urdu speaking	no	54	gg
control	24-28	male	72	5.6	25.5	B+	married	urdu speaking	no	65	AA
control	29-33	male	84	5.8	28	O+	married	baloch	no	69	AA
control	34-38	male	80	5.1	25.3	O+	married	urdu speaking	no	52	AA
control	29-33	male	79	5.1	24.8	O+	married	sindhi	no	75	AA
control	29-33	male	75	5.6	26.6	A+	married	sindhi	no	93	AA
control	29-33	male	75	5.1	23.7	A+	married	sindhi	no	78	AA
control	29-33	male	72	5.9	23.3	O+	married	sindhi	no	62	AA
control	24-28	male	85	5.1	26.8	A+	married	sindhi	no	68	AA
control	34-38	male	83	5.7	28.6	A+	married	sindhi	no	69	AA
control	18-23	female	67	5.4	25.2	A+	single	sindhi	no	79	AA
control	18-23	female	70	5	30.1	B+	single	punjabi	no	85	AA
control	24-28	male	85	5.9	27.6	B+	married	punjabi	no	70	Ag
control	24-28	male	70	6	20.9	B+	married	punjabi	no	97	AA
control	18-23	female	59	5.6	20.9	O+	married	punjabi	no	94	Ag
control	34-38	female	57	5	24.5	O+	married	pathan	no	79	Ag
control	18-23	female	60	5	25.8	A-	single	pathan	no	76	AA
control	24-28	male	70	5.11	21.5	B-	married	pathan	no	96	gg
control	24-28	male	75	5.9	24.4	A+	married	pathan	no	87	AA
control	24-28	male	82	5.7	28.3	B+	single	pathan	no	89	AA
control	24-28	male	90	5.8	30.1	O+	single	pathan	no	74	AA
control	24-28	male	76	5.1	24	0-	single	pathan	no	69	AA
control	24-28	female	70	5.4	26.4	AB+	married	pathan	no	95	AA
control	24-28	female	69	5.2	27.8	0-	married	baloch	no	75	AA
control	18-23	female	65	5.7	22.4	AB-	single	baloch	no	77	AA
control	18-23	female	55	5.2	22.1	O+	single	urdu speaking	no	88	AA
control	24-28	female	68	5.5	24.9	O+	single	urdu speaking	no	83	AA
control	24-28	female	82	5.8	27.4	B+	married	others	no	85	AA
control	24-28	female	85	5.7	29.3	B+	married	others	no	84	AA
control	34-38	male	95	5.1	30	B+	married	others	no	74	AA
control	34-38	female	60	5	25.8	O+	married	others	no	76	Ag
cases	24-28	male	79	6	23.6	O+	married	urdu speaking	yes	97	AA
cases	24-28	male	80	6.2	22.6	AB+	married	urdu speaking	yes	98	AA
cases	18-23	male	82	5.8	23.2	AB+	single	urdu speaking	yes	87	AA
cases	18-23	male	89	5.9	28.9	A+	single	punjabi	yes	84	AA
cases	24-28	male	73	5.8	24.4	A+	single	punjabi	yes	90	AA
cases	24-28	male	74	5	31.6	A+	single	punjabi	yes	96	AA
cases	34-38	male	69	5.9	22.4	B+	married	punjabi	yes	98	AA
cases	18-23	male	92	5.1	29	B+	single	punjabi	yes	70	gg
cases	18-23	male	84	5.8	28.1	O+	single	punjabi	yes	89	gg
cases	24-28	female	80	5.2	32.2	B+	married	punjabi	yes	82	gg
cases	24-28	female	82	5.5	30	B+	married	sindhi	yes	79	gg
cases	18-23	female	69	5.6	24.5	B+	married	sindhi	yes	70	gg
cases	34-38	male	76	5.8	25.4	B+	married	sindhi	yes	80	gg
cases	18-23	male	70	5.8	23.4	AB+	single	pathan	yes	87	gg
cases	29-33	male	75	5.8	25.1	A-	single	pathan	yes	86	gg
cases	29-33	male	76	5.9	24.7	A+	single	pathan	yes	86	gg
cases	29-33	male	78	5.9	25.3	B+	single	punjabi	yes	86	gg
cases	29-33	male	80	5.1	25.3	B+	married	punjabi	yes	81	AA
cases	24-28	male	81	5.1	25.5	O+	single	punjabi	yes	90	AA
cases	24-28	female	69	5.3	26.9	B-	married	punjabi	yes	90	AA
cases	24-28	female	60	5.2	24.1	O+	married	punjabi	yes	91	AA
cases	24-28	male	74	5.1	23.4	0-	married	urdu speaking	yes	93	AA
cases	24-28	male	78	6	23.3	A+	married	urdu speaking	yes	98	AA
cases	24-28	male	83	6.2	23.4	B+	married	urdu speaking	yes	99	AA
cases	29-33	male	84	6	25.1	B+	married	urdu speaking	yes	80	AA
cases	29-33	male	81	5.1	25.6	B+	married	urdu speaking	yes	74	AA
cases	18-23	male	80	5.9	26	A+	single	urdu speaking	yes	87	AA
cases	18-23	female	68	5.8	22.7	B+	single	urdu speaking	yes	89	AA
cases	18-23	female	69	5.4	26.1	A+	single	punjabi	yes	88	AA
cases	34-38	female	70	5.4	26.4	B+	married	punjabi	yes	76	AA
cases	34-38	male	71	5.9	23.1	O+	married	punjabi	yes	70	AA
cases	34-38	male	71	5.9	23.1	O+	married	punjabi	yes	72	AA
cases	29-33	male	60	5.6	21.3	O+	single	others	yes	65	AA
cases	18-23	male	70	5.8	23.4	B-	single	others	yes	69	AA
cases	24-28	male	86	5.9	27.9	O+	married	others	yes	67	AA
cases	18-23	male	97	5.4	36.6	B+	single	others	yes	96	AA
cases	24-28	male	98	5.9	31.8	B+	single	others	yes	85	gg
cases	24-28	male	73	6	21.8	A+	single	punjabi	yes	79	gg
cases	24-28	female	70	5.4	26.4	0-	married	punjabi	yes	69	gg
cases	24-28	female	77	5.4	26	A+	married	sindhi	yes	80	gg

**Table 5:** SPSS data for ANOVA SPSS: Statistical Package for the Social Sciences; BMI: body mass index; FBS: fasting blood sugar; ANOVA: analysis of variance

GENOTYPE	GROUP	AGE	BMI	FBS
aa	control	23	20.5	95
aa	control	22	17.6	98
aa	control	23	20	82
aa	control	28	20.7	82
aa	control	18	24.1	85
aa	control	18	21.1	93
aa	control	18	20.5	85
aa	control	20	22.9	84
aa	control	22	19	82
aa	control	23	28.9	71
aa	control	30	26.9	77
gg	control	22	22.9	92
gg	control	22	23	91
ag	control	22	20.8	64
gg	control	22	23.7	99
gg	control	23	20.1	86
ag	control	23	18.7	80
aa	control	23	21.4	90
aa	control	21	23	85
aa	control	32	32	81
aa	control	35	28.4	82
gg	control	22	17.2	85
gg	control	21	29.8	72
gg	control	27	14.5	85
gg	control	22	28.3	67
gg	control	22	18.9	79
gg	control	23	24.2	74
ag	control	26	20.1	54
aa	control	26	19.5	79
aa	control	20	19.5	78
aa	control	32	38.5	79
aa	control	22	19.5	63
aa	control	23	28.4	69
aa	control	34	25.5	64
gg	control	26	27.6	74
gg	control	23	20.3	98
gg	control	23	21	77
gg	control	34	29.3	89
gg	control	30	24.9	86
gg	control	30	27.6	98
gg	control	25	25.5	67
gg	control	30	31.1	68
gg	control	30	33.1	73
aa	control	31	21.1	100
gg	control	29	21.1	54
aa	control	25	25.5	65
aa	control	30	28	69
aa	control	34	25.3	52
aa	control	29	24.8	75
aa	control	32	26.6	93
aa	control	30	23.7	78
aa	control	30	23.3	62
aa	control	25	26.8	68
aa	control	34	28.6	69
aa	control	23	25.2	79
aa	control	23	30.1	85
ag	control	25	27.6	70
aa	control	27	20.9	97
ag	control	23	20.9	94
ag	control	34	24.5	79
aa	control	22	25.8	76
gg	control	22	21.5	96
aa	control	25	24.4	87
aa	control	25	28.3	89
aa	control	26	30.1	74
aa	control	26	24	69
aa	control	28	26.4	95
aa	control	28	27.8	75
aa	control	21	22.4	77
aa	control	22	22.1	88
aa	control	25	24.9	83
aa	control	26	27.4	85
aa	control	27	29.3	84
aa	control	34	30	74
ag	control	35	25.8	76
gg	cases	29	24.1	93
gg	cases	22	22.9	99
gg	cases	23	16.8	80
gg	cases	20	22.3	96
gg	cases	23	21.3	97
gg	cases	29	21.3	91
gg	cases	22	22.2	80
gg	cases	22	18.5	100
gg	cases	20	23.3	98
gg	cases	22	19.7	84
aa	cases	23	19.6	99
aa	cases	25	21.3	98
aa	cases	26	20.8	81
gg	cases	34	27	77
gg	cases	27	20.1	98
gg	cases	35	26.2	63
gg	cases	27	28.4	56
ag	cases	23	26	84
ag	cases	22	26	79
aa	cases	34	25.9	64
gg	cases	34	26.6	65
gg	cases	27	31.9	77
ag	cases	26	28.1	78
ag	cases	22	26.4	85
ag	cases	34	25.4	92
gg	cases	30	28.2	88
gg	cases	30	25.1	91
gg	cases	25	25.1	76
gg	cases	24	18.7	59
gg	cases	26	17.5	77
gg	cases	28	23	83
ag	cases	29	18	77
ag	cases	27	37.4	48
gg	cases	27	28.2	93
gg	cases	23	19.5	87
aa	cases	27	23.6	97
aa	cases	23	22.6	98
aa	cases	26	23.2	87
aa	cases	20	28.9	84
aa	cases	28	24.4	90
aa	cases	25	31.6	96
aa	cases	34	22.4	98
gg	cases	22	29	70
gg	cases	23	28.1	89
gg	cases	26	32.2	82
gg	cases	27	30	79
gg	cases	22	24.5	70
gg	cases	34	25.4	80
gg	cases	21	23.4	87
gg	cases	32	25.1	86
gg	cases	33	24.7	86
gg	cases	31	25.3	86
aa	cases	30	25.3	81
aa	cases	25	25.5	90
aa	cases	26	26.9	90
aa	cases	27	24.1	91
aa	cases	27	23.4	93
aa	cases	28	23.3	98
aa	cases	28	23.4	99
aa	cases	33	25.1	80
aa	cases	32	25.6	74
aa	cases	22	26	87
aa	cases	23	22.7	89
aa	cases	23	26.1	88
aa	cases	34	26.4	76
aa	cases	35	23.1	70
aa	cases	35	23.1	72
aa	cases	23	21.3	65
aa	cases	23	23.4	69
aa	cases	24	27.9	67
aa	cases	22	36.6	96
gg	cases	22	31.8	85
gg	cases	26	21.8	79
gg	cases	25	26.4	69
gg	cases	26	29	80

**Table 6:** 18-23 age frequency separately We have shown the age range because the majority group we have taken to perceive the genetic role belonged to the 18-23 age group. We have attached the frequency file.

Column1	AGE	Frequency	Percent	Valid Percent	Cumulative Percent
Valid	18.00	3	2.0	2.0	2.0
	20.00	5	3.3	3.3	5.3
	21.00	4	2.7	2.7	8.0
	22.00	24	16.0	16.0	24.0
	23.00	24	16.0	16.0	40.0
	24.00	2	1.3	1.3	41.3
	25.00	12	8.0	8.0	49.3
	26.00	14	9.3	9.3	58.7
	27.00	12	8.0	8.0	66.7
	28.00	7	4.7	4.7	71.3
	29.00	5	3.3	3.3	74.7
	30.00	11	7.3	7.3	82.0
	31.00	2	1.3	1.3	83.3
	32.00	5	3.3	3.3	86.7
	33.00	2	1.3	1.3	88.0
	34.00	13	8.7	8.7	96.7
	35.00	5	3.3	3.3	100.0
	Total	150	100.0	100.0	
